# Influence of deep eutectic solvents on redox biocatalysis involving alcohol dehydrogenases

**DOI:** 10.1016/j.heliyon.2024.e32550

**Published:** 2024-06-06

**Authors:** Ebin K. Baby, Rangasamy Savitha, Gemma K. Kinsella, Kieran Nolan, Barry J. Ryan, Gary T.M. Henehan

**Affiliations:** a School of Food Science and Environmental Health, Technological University Dublin, Grangegorman Lower, Dublin 7, D07 E244, Ireland; b School of Chemical Sciences, Dublin City University, Glasnevin, Dublin 9, D09 V209, Ireland

**Keywords:** Deep eutectic solvents, Alcohol dehydrogenases, Solvent engineering, Biocatalysis, Enantioselectivity

## Abstract

Redox biocatalysis plays an increasingly important role in modern organic synthesis. The recent integration of novel media such as deep eutectic solvents (DESs) has significantly impacted this field of chemical biology. Alcohol dehydrogenases (ADHs) are important biocatalysts where their unique specificity is used for enantioselective synthesis.

This review explores aspects of redox biocatalysis in the presence of DES both with whole cells and with isolated ADHs. In both cases, the presence of DES has a significant influence on the outcome of reactions albeit via different mechanisms. For whole cells, DES was shown to be a useful tool to direct product formation or configuration - a process of solvent engineering. Whole cells can tolerate DES as media components for the solubilization of hydrophobic substrates. In some cases, DES in the growth medium altered the enantioselectivity of whole cell transformations by solvent control. For isolated enzymes, on the other hand, the presence of DES promotes substrate solubility as well as enhancing enzyme stability and activity. DES can be employed as a smart solvent or smart cosubstrate particularly for cofactor regeneration purposes.

From the literatures examined, it is suggested that DES based on choline chloride (ChCl) such as ChCl:Glycerol (Gly), ChCl:Glucose (Glu), and ChCl:1,4-butanediol (1,4-BD) are useful starting points for ADH-based redox biocatalysis. However, each specific reaction will require optimisation due to the influence of several factors on biocatalysis in DES. These include solvent composition, enzyme source, temperature, pH and ionic strength as well as the substrates and products under investigation.

## Highlights

• Deep eutectic solvents (DES) can dramatically alter biocatalytic processes.
• DES can influence the enantioselectivity of chiral synthesis in whole cells.
• DES can enable enhanced reaction yields by enhancing enzyme performance (stability, activity, specificity).
• DES components can be a useful cosubstrate in cofactor regeneration.

## Graphical abstract

**Figure:**
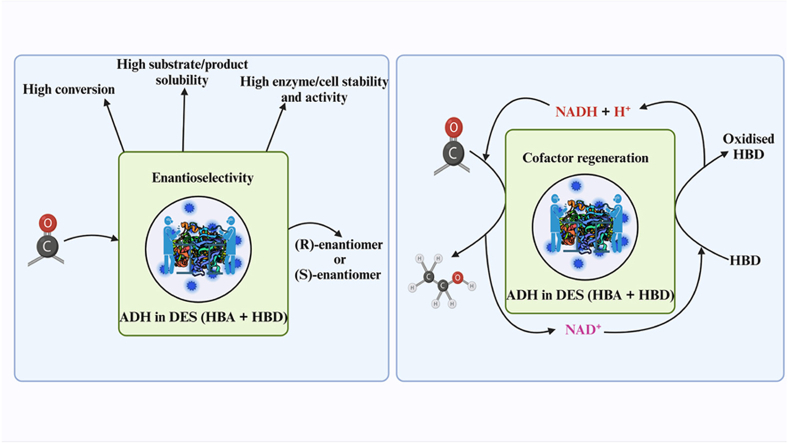

## 1 Introduction

The paradigm of sustainable and green chemistry introduces a new perspective to the fields of chemistry and chemical engineering. Sustainability principles have evolved over time, guiding the design, development, and implementation of chemical products and processes [1]. These principles empower scientists and engineers to actively contribute to the well-being of the economy, humanity, and the planet [2]. A key focus is the development of innovative approaches to minimise waste, conserve energy, and identify substitutes for hazardous substances [3]. The utilisation of inorganic chemicals, organic reagents/solvents, the subsequent downstream processes and the use of complex multi-step synthesis procedures contribute to waste generation in chemical manufacture. A potential solution to this challenge is the integration of biocatalysts (mostly enzymes) into chemical processes [4,5]. Biocatalysis can enable the production of products that are not readily achievable through conventional chemical synthesis. They may also use alternative, and cheaper, raw materials and thus reduce operational costs. Moreover, they often require minimal fixed infrastructure [6]. Some biocatalysts have demonstrated operational stability in organic solvents and this opens up scope for biocatalysts in organic synthesis [7]. However, using organic solvents for certain reactions reduces the environmental desirability of this type of biocatalysis. Considering this shortcoming, the use of modern solvents, such as ionic liquids (IL) [8,9] and more recently DES [10], in biocatalysis is one of the major breakthroughs in improving the sustainability of organic synthesis (see Supplementary Information Fig. S1). Additionally, DESs, when used as a solvent or cosolvent, can enhance the solubility of hydrophobic substrates. For instance, the introduction of DESs has improved the solubility of halogenated hydrocarbons, epoxides, and several drugs, while also reducing unwanted reactions. DES can help to enhance the yield, stability, and, in many cases, the activity of enzymes [[10], [11], [12], [13], [14], [15], [16]]. In some cases, DES can influence the enantioselectivity of reactions, adding to their growing importance [[17], [18], [19], [20]]. This review largely considers the reduction of carbonyl compounds to yield chiral alcohols through ADH mediated reactions, an example of one of the key enantioselective reactions that play a central role in organic synthesis [21]. This enantioselective ability, makes ADH a powerful, and industrially significant biocatalyst [22].

### 1.1 Alcohol dehydrogenase: a powerful redox biocatalyst

ADH, is a fully reversible alcohol oxidising enzyme that is capable of alcohol oxidation and ketone/aldehyde reduction as well as aldehyde oxidation/dismutation (see Table 1).

**Table 1:** The variety of reactions catalysed by ADHs.

No.	Reaction	Scheme	Ref.
1	Alcohol oxidation		[23]
2	Carbonyl reduction		[[24], [25], [26]]
3	Aldehyde oxidation		[27]
4	Aldehyde dismutation		[[28], [29], [30], [31]]

*Note*: direct aldehyde oxidation to the carboxylic acid is normally only observed under NADH recycling conditions when NADH concentrations are low. Prior hydration of the aldehyde is shown (reaction 3) for aldehyde oxidation. The hydration produces a *gem-diol* which is structurally analogues to a secondary alcohol. The oxidation of the hydrated aldehyde to form a carboxylic acid is not reversible.

ADH has a wide range of applications [32,33], and has been utilised in diverse multi-step cascades to transform simple starting materials into complex products [22,34,35]. For instance, beginning with a cyclic ketone and diol, a redox-neutral ADH-monooxygenase cascade for producing lactones has been demonstrated [36].

**Fig. 1:**
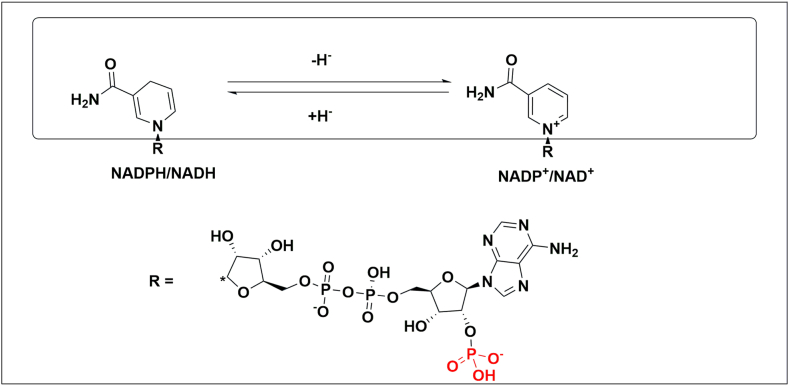
Nicotinamide adenine dinucleotide non-phosphorylated (NADH/NAD^+^) and its phosphorylated ribose (NADPH/NADP^+^) form. The adenine dinucleotide portion of the molecule is indicated as R. The site of phosphorylation, on the ribose moiety, is highlighted in red. The reversible transfer of a hydride ion is indicated. Note that the nicotinamide portion of the NAD(P) is the recipient or donor of a hydride ion during redox catalysis.

Redox biocatalysis requires a cofactor to complete the redox cycle. The cofactor is reduced, or oxidised, according to the reaction type [22,37]. There are two types of cofactors for ADH, nicotinamide adenine dinucleotide non-phosphorylated (NADH/NAD^+^) and its phosphorylated form (NADPH/NADP^+^; see Fig. 1), with both forms presenting similar physiochemical properties [22].

A major challenge in the synthetic application of ADHs is cofactor regeneration; cofactors are expensive and consequently, regeneration is necessary [38]. This limitation has been addressed by using whole cells, as opposed to an isolated (purified) enzyme, as the biocatalyst source. Early studies with purified enzymes concentrated on ADHs from horse liver [32] and yeast [39] but in recent years a number of other ADH have been employed. A selection of purified ADH enzymes used in biocatalysis is shown in Table 2. In addition, a variety of ADHs used in whole cells have been described [10].

**Table 2:** Some ADHs used in biocatalysis and their structural features.

Enzyme	Structural Features	Ref.
Yeast ADH	Medium chain, tetramer, 347 amino acid residues per polypeptide chain and contains a catalytic zinc ion	[39,40]
*Thermoanaerobacter brockii* ADH	Medium chain, tetramer, 352 amino acids residues per polypeptide and contains a catalytic zinc ion	[40]
Horse liver ADH	Medium chain, dimer, 374 amino acid residues per polypeptide chain and contains a catalytic zinc ion	[39,40]
*Candida parapsilosis* ADH	Medium chain, dimer, 279 amino acids per polypeptide and contains a catalytic zinc ion	[41]
Drosophila ADH	Short chain, dimer, 250 amino acid residues per polypeptide chain, no metal ion	[40]
*Clostridium beijerinckii* ADH	Medium chain, tetramer, ∼351 amino acid residues per polypeptide and contains a catalytic zinc ion	[40]
*Pyrococcus furiosus* AdhD	Short chain, monomer with 278 amino acids, no metal ion	[42]
*Entamoeba histolytica* ADH2	Medium chain, tetramer, 453 amino acids residues per chain and contains iron	[43,44]
*Geobacillus thermodenitrificans* ADH2	Long chain, homodimer with 387 amino acids, contains iron	[45,46]
*Geobacillus thermodenitrificans* ADH1	Long chain, homooctamer with 395 amino acids, contains iron	[45,46]
*Sphingobium yanoikuyae* ADH	Short chain, 262 amino acids	[47]
*Ralstonia* ADH	Short chain, trimer in solution, 249 amino acids	[48]

Whole cell biocatalyst sources are often favoured over the use of isolated enzymes due to their affordability since using whole cells directly reduces the production and purification cost of isolating an enzyme [20]. Despite these advantages, whole cell approaches come with drawbacks, such as, the formation of side products, demanding additional downstream steps in product isolation/purification, and potential cell toxicity induced by substrates/products [49]. To fully appreciate the use of ADH in redox biocatalysis, it is important to understand these factors and the mechanism of ADH-catalysed reactions.

### 1.2 The mechanism of ADH catalysed reactions

**Fig. 2:**
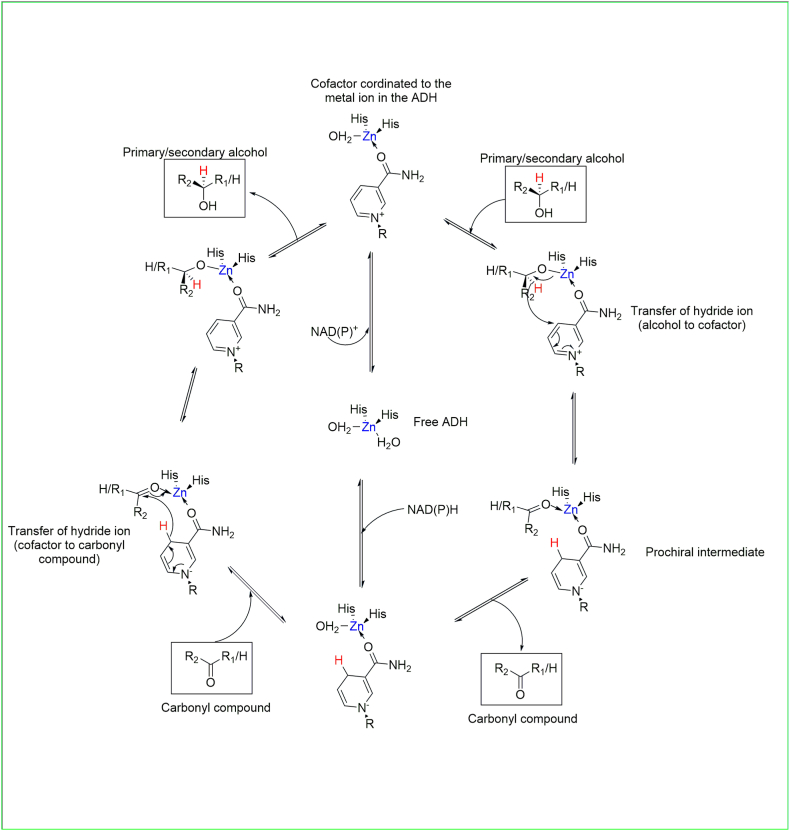
Mechanism of ADH catalysed reduction and oxidation involving an active site metal (zinc). The nicotinamide cofactor is shown coordinated to the active site zinc. The binding of the alcohol compound leads to hydride transfer to the nicotinamide moiety of NAD^+^ and the binding of the carbonyl compound (reverse reaction) leads to the abstraction of hydride ion from the nicotinamide moiety of NADH. Note: The R group attached to the nicotinamide represents the remainder of the cofactor (see Fig. 1). R_1_ and R_2_ represent alkyl or aryl substituents. The hydride ion transferred during the redox reaction is highlighted in red. Adapted from Ref. [22].

ADHs tend to be broad specificity enzymes capable of binding a range of aliphatic and aromatic alcohols/carbonyls. The reaction is fully reversible, and the enzyme can reduce aldehydes or ketones to the corresponding alcohol (see Table 1). A detailed mechanism of a catalytic zinc-containing ADH is shown in Fig. 2. The catalytic activity of the ADH is initiated by the binding of cofactor [NAD(P)^+^/NAD(P)H] to the enzyme surface followed by the binding of substrate (alcohol or carbonyl compound). Hydride ion transfer occurs between cofactor and substrate [22].

**Fig. 3:**
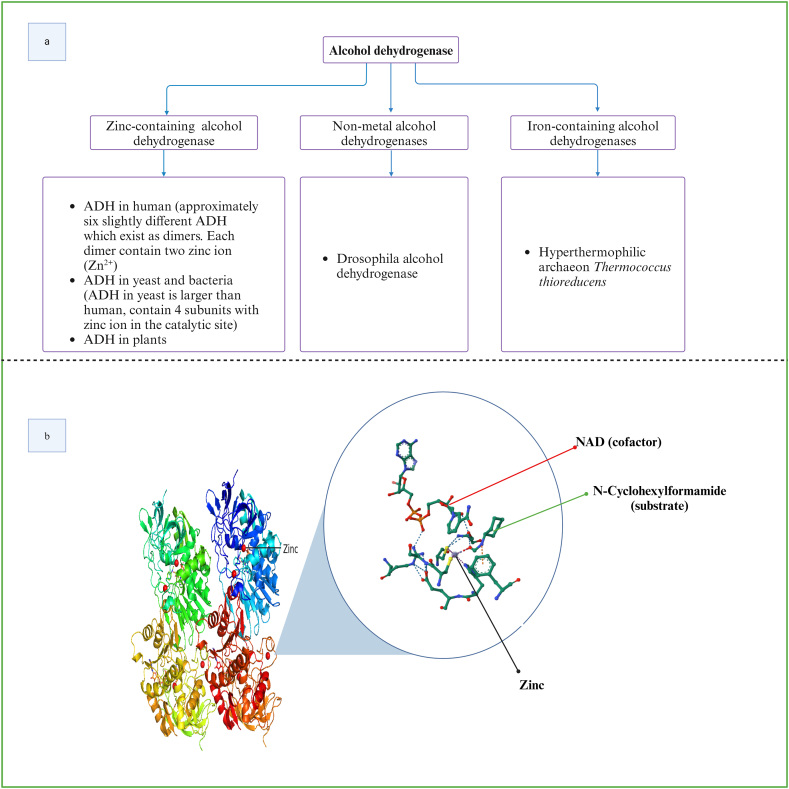
a) Classification of ADH based on the metal ion in the active site [39,40,53] from different species. b) 3D structure of horse liver ADH containing zinc ion, inset shows the zinc ion complexed to NADH and cyclohexyl formamide (PDB code-1LDY) [54]. Created with BioRender.com.

Many ADHs exist as metalloenzymes (See Fig. 3a and b), and in these cases a metal ion (e.g. zinc or iron ) is present in their active sites [[50], [51], [52]]. The metal ion assists in the coordination of both the cofactor and the substrate keeping them in close proximity such that the hydride ion transfer becomes more favourable [22].

**Fig. 4:**
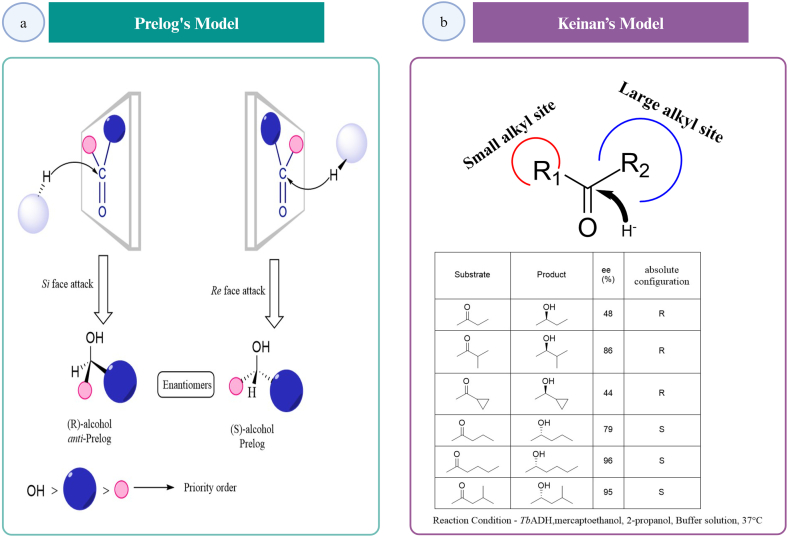
Stereochemical recognition in asymmetric reduction reactions catalysed by ADHs. a) Prelog model, which was based on the addition/transfer of hydride ion (H^−^) to/from the cofactor to the substrate giving corresponding (R) alcohol (*anti-*Prelog ADH)/(S) alcohol (Prelog ADH) or the carbonyl compound [55]. b) Keinan model to explain enantiomeric outcomes in ADH-catalysed reactions which was based on the bulkiness of groups in the space surrounding the carbonyl moiety: above a certain bulk, some alkyl groups cannot bind to a smaller site (occupied by R_1_ above) and will enter the larger site leading to the observed transition from R to S configuration [56]. Created with BioRender.com.

There are four possibilities to transfer the hydride ion from the cofactor to the substrate in the reduction of a prochiral ketone. Two *re* (back) face transfer and two *si* (front) face transfer of the hydride ion [22]. ADH can be Prelog-categorised as Prelog and *anti*-Prelog ADH, based on the transfer of the hydride ion [55]. If an ADH adds a hydride ion to the *si* face of the prochiral ketone to produce an (R)-alcohol, or if the ADH abstracts protons from (R)-alcohols, it is referred to as an *anti*-Prelog ADH. Whereas, if the addition of the hydride ion occurs through the *re* face to form (S)-alcohol, or the ADH abstracts the hydride ion from (S)-alcohols, then the ADH is termed as Prelog ADH (see Fig. 4a) [22,55]. Alternatively, Keinan and colleagues explained the stereoselectivity of *Tb*ADH for the reduction of ketones, as being modulated by large and small alkyl binding sites on the enzyme surface. Above a certain bulk, the smaller alkyl site was unoccupied causing enantiomeric inversion. When the small alkyl groups were methyl, ethyl, isopropyl or cyclopropyl, the hydride transfer occurred through the *si* face of the substrate giving an (R)-alcohol product. This was not the case with *n*-propyl and bulkier groups where inversion of configuration occurs (see Fig. 4b) [56].

### 1.3 Modulating ADH catalysed reactions for enantioselectivity

With the different hydride transfer possibilities, ADH catalysis is enantioselective [57]. Recent studies have shown that the reaction can be judiciously manipulated by altering the reaction conditions, such as varying the solvent to yield excess R or S alcohol products from corresponding ketones [58,59]. Along with solvent, temperature and pH, play a significant role in achieving the desired enantiomeric conversion [22,37,[60], [61], [62], [63], [64]].

In chemical kinetics, the rate of a chemical reaction doubles with every 10 °C rise in temperature [65]. However, this is not the case with biocatalysis where there is a trade-off between activity and enzyme stability at higher temperatures [66,67]. The optimal temperature for an enzyme is the intersection point of the activity (increasing) curve and the stability (decreasing) curve [22,61].

The effect of pH on organic synthesis by ADHs often depends on whether oxidation or reduction is considered. The reduction of carbonyl compounds is favoured in slightly acidic conditions while the oxidation of alcohols is favoured in alkaline conditions [68]. However, changing the pH of the solution significantly may affect the enzyme's conformation and activity by changing the state of ionisation of catalytic amino acids [67]. Isolated ADH is generally highly active at neutral pH; however, the reduced form of the cofactor (NAD(P)H) is most stable in an alkaline medium and its oxidised form is most stable in an acidic medium [62].

A prominent consideration in biocatalysis is the nature of the solvent(s) used for substrate solubilization and their effect on the enzyme(s). Recent advances in conventional organic, and neoteric solvents require careful consideration of the variation in ADH activity in the chosen solvent system [63]. Thus, enantiomers formed in certain reactions may depend on the composition of the solvent and it has been reported that DES can dramatically alter enantioselectivity [25]. This approach of enhancing enantioselectivity is an example of solvent engineering in biocatalysis [10,[18], [19], [20]].

Given that this is a significant area of rapid development, this review focuses specifically on the influence of DESs on ADH reactions either, (i) as catalysed via whole cell-based ADH, or (ii) catalysed by isolated cell-free enzymes. Much of the focus is on enantioselective reduction reactions, the main application area for ADH. This review also considers the strategies for cofactor regeneration in DES.

## 2 Deep eutectic solvents and whole cell biocatalysis

DESs are a special category of eutectic mixture, which are typically created between hydrogen bond donors (HBDs) and hydrogen bond acceptors (HBAs) or mixtures of Lewis acids and bases. Widely used HBDs are urea, glycerol, ethylene glycol, glucose and widely used HBAs are ammonium or phosphonium-based salts. An essential criterion is that the acidity difference between the components of DES should be minimal [69]. They are prepared either by mixing the components with gentle heating and stirring or by mixing components in a common solvent followed by solvent removal. This ease of preparation is an advantage of DES in comparison to ionic liquids [69]. Perhaps surprisingly a number of cell types (e.g. cultured plant cells, microbial cells) showed improved cell permeability, stability and reduced cytotoxicity in the presence of DES [64,[70], [71], [72], [73]]. For example, whole *Cyberlindnera saturnus* cells have been used for the synthesis of (*S*)-1-[3,5 bis(trifluoromethyl)phenyl]ethanol [(*S*)- BTPE] from 3,5-bis(trifluoromethyl)acetophenone (BTAP) in DES, l-carnitine:lysine (C:Lys). The introduction of DES (C:Lys) increased cell permeability to substrate and reduced the substrate (BTAP) toxicity [73]. Thus, the reduction of carbonyl compounds by whole cells in the presence of DES offered significant advantages over aqueous media. The examples given below are for ketone reduction to chiral alcohols where there is evidence that the main enzyme involved in the whole cell transformation is an ADH.

### 2.1 Whole cell carbonyl reductions in deep eutectic solvents

Numerous industrially relevant processes require alcohol functionalities. Reductions, particularly the reduction of prochiral ketones into their corresponding chiral alcohols, are an important reaction in organic chemistry [22,35]. Such reactions are efficiently performed through biocatalysis. By the end of the 20th century, the use of traditional biocatalytic reactions had transitioned from the use of organic solvents to ionic liquids (IL). More recently, the move to the use of DES, and the combination of DES with IL, has gained ground. For example, a biphasic medium of DES [ChCl:ethylene glycol (EG)] and IL [1-butyl-3-methylimidazolium hexafluorophosphate (C_4_MIM·PF_6_)] was used to reduce 2-octanone to its corresponding (R)-alcohol with the help of *Acetobacter pasteurianus* Gim1.158 cells (see Supplementary Information Table S1). The addition of IL to this DES system increased the solubility of both product and substrate as well as the operational stability of the whole cell. The DES (ChCl:EG) showed good biocompatibility and served to increase cell permeability [11]. In general, the reaction yield is contingent on the chosen DES and, therefore, careful selection of DES is pivotal [10,18]. For example, different DESs were tested for the reduction of 2-hydroxyacetophenone (HAP) to (R)-1-phenyl-1,2-ethanediol (PED) by using *K. gibsonii* SC0312 cells [70]. Here, the use of ChCl:1,4-BD provided good yield (80 %) in conjunction with good cell biocompatibility in comparison to other DESs tested [ChCl:urea (U), ChCl:triethylene glycol (TEG), ChCl:EG, ChCl:Gly (details in Supplementary Information Table S2)]. This system, 80 mM HAP in ChCl:1,4-BD yielded 80 % (R)-PED with an optical purity >99 % [70].

**Scheme 1:**
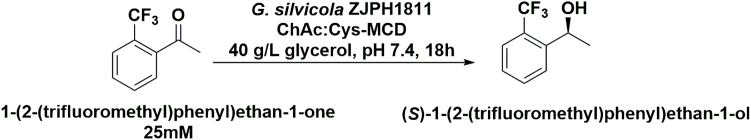
Asymmetric reduction of 1-(2-(trifluoromethyl)phenyl)ethan-1-one with *G. silvicola* ZJPH1811. ee is enantiomeric excess.

DESs can also be prepared using a variety of oligopeptides and amino acids (Supplementary Information Tables S3, S4, S5). For instance, comparing amino acid and oligopeptide-based DESs [ChCl with glutathione (GSH)], an oligopeptide-based DESs (ChCl:GSH) demonstrated an enhanced yield (from 70.4 % to 87.6 %) for the reduction of BTAP to the corresponding (R)-BTPE using *T. asperellum* ZJPH0810 cells (see Supplementary Information Table S3) [71]. However, by using the same substrate (BTAP) with the yeast isolate *Cyberlindnera saturnus* ZJPH1807 in C: Lys in the presence of Tween-80, a different enantioselectivity was favored producing (S)-BTPE (see Supplementary Information Table S6) [73]. It should be emphasised that Tween 80, a surfactant, is utilised in the reaction in conjunction with the DES. Tween 80 is also used as a surfactant in the synthesis of (S)-4-chloro-3-hydroxybutanoate [(S)-CHBE]. The conversion of ethyl 4-chloro-3-oxobutanoate (COBE) to (S)-CHBE was conducted with recombinant *E. coli* CCZU-T15 cells in the presence of Tween 80 and a ChCl:Gly-water mixture. Here an enantiomeric excess (ee) of >99 % was obtained (see Supplementary Information Table S7) [74]. In short, the use of a surfactant can increase the dispersion of water-insoluble substrates through the formation of a micellar system in DES, increasing substrate mass transfer and, therefore, product yield [73,74]. The same strategy was employed in the reduction of 2-chloro-1-(2,4-dichlorophenyl) ethanone (CPE) to (R)-2-chloro-1-(2,4-dichlorophenyl)ethanol [(R)-CPEO] using *C*. *saturnus* ZJPH1807 cells in C:trehalose (Tre), supplemented with cyclodextrin (CD) (see Supplementary Information Table S8) [75]. Similarly, for the transformation of 2ʹ-(trifluoromethyl) acetophenone by *G. silvicola* in choline acetate (ChAc):Cys, the addition of methylated-β-cyclodextrin (MCD) serves a comparable role (see Scheme 1) [76].

CD and its derivatives, akin to Tween 80, enhance substrate solubility and can boost yield [75,76]. For instance, in the conversion of 2ʹ-(trifluoromethyl) acetophenone, the yield in ChAc:Cys increased from 72.3 % to 83.2 % with the addition of MCD [76]. The addition of organic solvent to DES can also increase the substrate and product solubility. For example in the biotransformation of COBE into (R)-CHBE with *E*. *coli* CgCR, a high (R)-CHBE yield of 97.6 % was obtained in the bioreaction system containing 7 % (v/v) betaine:lactic acid (B:LA) and 50 % (v/v) ethyl acetate [77].

However, there are certain challenges when using DESs, primarily due to their inherent viscosity and cytotoxicity [78]. To overcome this, the inclusion of a cosolvent (buffer/water) is often necessary. For example, maximum enantioselectivity was achieved in ChCl:Gly with a 30 % (v/v) water system during asymmetric reduction of 1-(3,4- dimethylphenyl) ethenone using *S. cerevisiae* [Baker's Yeast (BY)], details provided in Supplementary Information Table S9 [79]. Moreover, the reaction yield was also dependent on the HBA chosen. In the initial studies on DES in biocatalysis, ChCl was commonly employed as the HBA [[80], [81], [82]]. However, each whole cell system exhibits distinct behavior with different solvents. It was found that at high concentrations of ChCl, HLADH activity diminishes [25]. Thus, it is important to assess a variety of DESs to identify the optimal solvent system for a given enzymatic reaction. In comparing DESs based on ChAc and ChCl as HBAs, enzyme activity was notably more efficient in ChAc when amino acids were used as HBDs in the bioconversion of 2-chloro-1-(3,4-difluorophenyl) ethanone (CFPO) into (S)-2-chloro-1-(3,4-difluorophenyl) ethanol [(S)-CFPL] using recombinant *E. coli* resting cells (see Supplementary Information Table S10) [78]. The most favourable results were achieved using ChAc:Lys, with an ee >99.99 %. Additionally, the inclusion of ChAc:Lys significantly improved cofactor regeneration, outperforming ChCl-based DESs. Here, optimal conditions were established at a temperature of 30 °C and a pH below 7.6, with DES concentrations less than 1 % (w/v). Above 1 % (w/v) DES concentration, the yield decreases, possibly due to increased cytotoxicity [78]. ChAc demonstrated greater efficiency than ChCl which has been attributed to the cytotoxicity of ChCl, induced by the chloride ion binding to the cell plasma membrane [78]. In many acid-based DESs, enzyme activity typically decreases e.g.; ChCl:oxalic acid (OA), ChCl:malic acid (MA) [83]. Recently, it has been discovered that acidic DESs can enhance bioreduction. In the conversion of CFPO to (S)-CFPL using recombinant *E. coli* cells containing the NADH-dependent reductase (CmCR) in 7.5 %, v/v ChCl:LA DES [84] instead of ChAc:Lys [78], substantially higher yield (90.8 %) was obtained, marking 1.4-fold increase over a purely aqueous culture medium. The reason suggested for the improved yield is the enhanced cell membrane permeability facilitated by the low viscosity of ChCl:LA (7.5 % v/v) (Supplementary Information Table S11) [84].

**Scheme 2:**
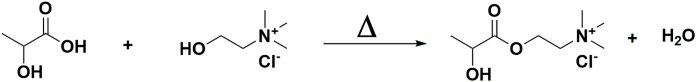
Ester formation in carboxylic acid-based DES during preparation and long-term storage.

The stability of DESs is significantly influenced by the preparation parameters and the application under consideration. For example, during the preparation of ChCl:LA, 2 mol% choline was found to be converted to a lactic acid choline ester at 60 °C (see Scheme 2). This increases to 4 mol% at 80 °C and 7 mol% at 100 °C. Long-term storage can increase the ester formation [85]. This stability issue is not limited to ChCl:LA, but it was also observed with other DES components such as malonic acid, glycolic acid, and levulinic acid [85]. For instance, malonic acid can decompose into acetic acid and carbon dioxide in DES [85]. In glucose-based DES, caramelization of glucose can occur at temperatures between 100 °C and 120 °C [86]. These degradation aspects are significant since most DESs preparation procedures involve heating at above 60 °C to achieve a homogeneous solution [85]. The impact of these degradation products on enzyme stability and activity remains unknown and requires further study.

Another important aspect to consider is the pH associated with the DES, which can vary with temperature. For example, the pH of ChCl:F (d-fructose) solution appears to decrease as the temperature increases. At 25 °C, the pH of ChCl:F (1:1) solution was 6.1 [87]. It is well known for ADHs that the reaction rates for alcohol oxidation increases with increasing pH up to pH 9–10, beyond which they decline [88]. In most cases, the fluctuating pH of reactions is controlled using buffer as component of the reaction mixture [25].

DESs are often mixed with water or an aqueous buffer in various ratios during their application. It is essential to understand DES can retain its properties after mixing with water up to a certain concentration [12,89]. For example, ChCl:U can maintain its nanostructural properties in up to 50 % w/w water [90]. ChCl:Gly (1:2) maintains its properties up to 35 % w/w water; at higher water levels, DES solutions become an aqueous electrolyte mixture [91]. The same principles apply to other DESs under consideration. Another critical factor is the water activity in DESs. A certain amount of free water is necessary for proper enzyme function. For instance, HLADH remains active in systems containing free water, while its activity decreases in neat DESs [25].

Other than choline derivatives, betaine (B), l-proline (P), l-carnitine (C), cetyltrimethylammonium bromide, and FeCl_3_ can be used as HBAs in DES formulations (see Table 3) [73,[92], [93], [94]].

**Table 3:** List of (NA)DES having a HBA other than ChCl [92] where B is Betaine, P is Proline, C is carnitine, Gly is Glycerol, Glu is Glucose, Lys is Lysine and G is Glycine.

No.	DES	Molar ratio
1	B:Glu:Gly	1:1:2
2	P:Glu:Gly	1:1:2
3	C:Glu:Gly	1:1:2
4	B:Lys	1:2
5	P:Lys	1:2
6	C:Lys	1:2
8	B:G	1:1

l-carnitine based DES (C:Lys) was found to be efficient for the reduction of 2,6-dichloro-3-fluoroacetophenone (see Supplementary Information Table S12) [92]. A betaine-based DES (B:Lys) was found to be efficient in several biocatalytic reductions as in the case of the reduction of 4-(trifluoromethyl) acetophenone by *G. geotrichum* ZJPH1810 cells and also with recombinant *E. coli* (see Supplementary Information Tables S13 and S14) [92]. Betaine based DES as an efficient solvent in the synthesis of 2,5-furandimethanol (FDM) from 5-hydroxymethylfurfural (see Scheme 3) has been reported [[95], [96], [97]].

**Scheme 3:**
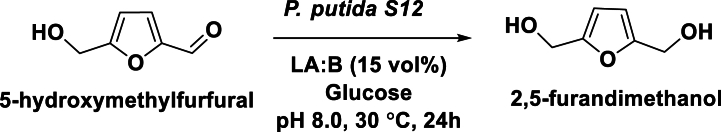
Synthesis of FDM from 5-hydroxymethylfurfural by *P. putida* S12 cells in betaine based DES.

**Scheme 4:**
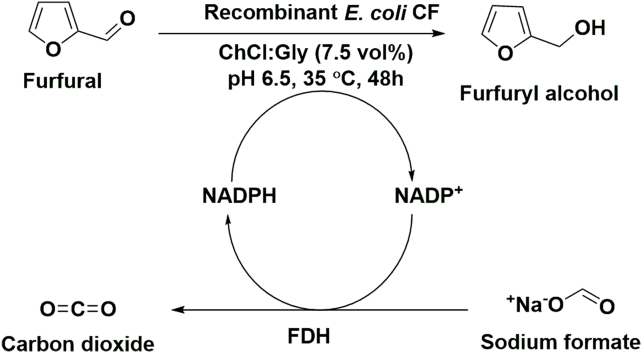
Synthesis of furfuryl alcohol from furfural with recombinant *E. coli* CF containing a reductase and cofactor recycling by formate dehydrogenase (FDH).

FDM is widely used in resin industries, in the production of synthetic fibres and fine chemicals [98]. ChCl:Gly (7.5% v/v) was found to be efficient in the synthesis of furfuryl alcohol from furfural [99] (see Scheme 4).

The use of plant cells, such as carrot and sugar beet, in DES is gaining attention in ADH-based reductive biocatalysis [64,79]. For example, cultured carrot roots were employed in the biocatalytic reduction of 1-(3,4-dimethylphenyl) ethenone (see Supplementary Information Table S15) [64]. Although superior conversion was achieved in pure aqueous solution, ChCl with glucose (Glu) as HBD demonstrated the highest conversion among the different DESs investigated. ChCl with EG showed the lowest conversion [64].

The activity of ADH sourced from different species may be different in the same DES. In the case of *Thermoanaerobacter ethanolicus* ADH (TeSADH), horse liver ADH (HLADH), and *Ralstonia* sp. ADH (RasADH) which were overexpressed in *E.coli;* each showed different activity in a ChCl:Gly (1:2) DES system. Here, the activity of TeSADH and HLADH was influenced by the concentration of DES, as the activity for both enzymes declined with increasing DES concentration above 90 % (v/v). However, RasADH was active at high DES levels: at a DES concentration of 95 % (v/v) (ChCl:Gly), half of the initial activity persists (see Supplementary Information Table S16) [100].

**Scheme 5:**
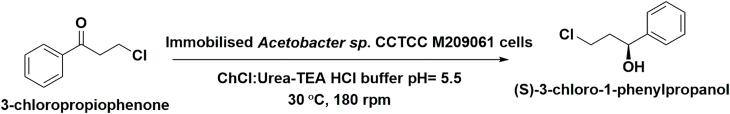
Asymmetric reduction of 3-chloropropiophenone with immobilised *Acetobacter* sp. CCTCC M209061 cells.

While the capabilities of whole cells in suspension is acknowledged, the potential application of immobilised whole cells is an emerging field with significant potential. This is because immobilised cells offer several advantages over free cells. These include simplified separation from the reaction medium, enhanced substrate conversion, reduced inhibition by products, shorter reaction times, and greater control over cell replication [101]. For instance, immobilised *Acetobacter* sp. CCTCC M209061 cells were used for the bioreduction of 3-chloropropiophenone to (S)-3-chloro-1-phenylpropanol in ChCl:U. DES showed biocompatibility with the cells and increased cell permeability and stability (see Scheme 5) [102].

As these reductions of this prochiral ketone can give a route to the corresponding enantiomers, careful adjustment of DES can selectively produce a desired enantiomer [83,100].

### 2.2 Deep eutectic solvent engineering to enhance the enantioselectivity of carbonyl reduction

**Scheme 6:**
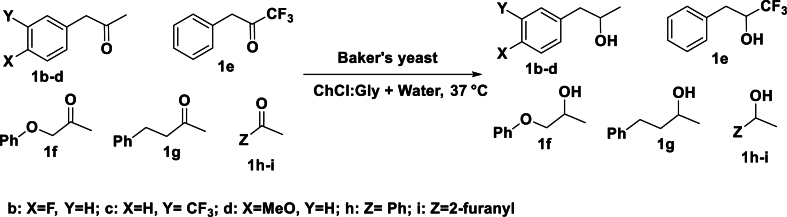
Enantioselective reduction of different ketones with Baker's yeast. The enantioselectivity varies with the reaction conditions [59].

An enantioselective reaction is a chemical reaction that produces predominantly one enantiomer of a chiral compound over the other [103]. The distinctive stereochemistry of each enantiomer underscores the significance of enantioselective reactions [57]. For example, selection of appropriate enantiomers can reduce the side effects and enhance the therapeutic efficacy of (bio)pharmaceutical compounds [104]. Beyond (bio)pharmaceuticals, enantioselective reactions are crucial across a spectrum of industries, encompassing, agrochemicals, flavour, fragrance, materials science and beyond [10,22,105]. Stereoselectivity can be tuned by judiciously adjusting the (NA)DES composition. NADES are modified DES which incorporate natural components as HBA and HBD (e.g. B:Lys) and these provide a cell-like environment to the whole cell (see Supplementary Information Fig. S1) [92]. For example, utilisation of BY in the bio-reduction of ethyl acetoacetate (EAA) in the presence of DES showed such stereoselectivity. Here, the reactions in pure water yielded the (S)-alcohol, [(S)-ethyl 3-hydroxybutyrate (EHB); ee > 95 %] whereas, using ChCl:Gly (1:2) resulted in (R)-alcohol, [(R)-ethyl 3-hydroxybutyrate; ee > 95 %]. A clear stereo-inversion occurred in the product while using pure water versus DES >80 % v/v and a racemic mixture was obtained in DES:water (80:20) (see Supplementary Information Table S17) [58]. For the bio-reduction of several arylpropanones with BY in DES, water, and DES-water mixtures, showed a similar stereo-inversion. When water was used as the solvent, it resulted in synthesis of excess (S)-alcohol (96 % ee); however, employing DES (ChCl:Gly) resulted in excess (R)-alcohol (96 % ee; see Scheme 6) [59].

In the two examples mentioned above, BY in ChCl:Gly primarily yielded (R)-alcohols. It is important to note that BY in ChCl:Gly does not consistently exhibit (R) selectivity, as the formation of (S)-alcohol was observed during the reduction of aryl ketones with BY cells in water and ChCl:Gly mixtures (20 w% water; see Supplementary Information Table S18) [106]. Thus, the DES, the whole cells used, and the nature of substrate are the important factors which influence the enantioselectivity of biocatalytic reaction [56].

Enantioselective enrichment has also been observed in reductions involving whole cells derived from plants. In the reduction of the prochiral ketone, 1-(3,4-dimethylphenyl) ethanone to its chiral, (1R)-1-(3,4-dimethylphenyl) ethanol using sugar beet (*Beta vulgaris* L. subsp. *vulgaris*), the highest enantioselectivity for the (S)-alcohol was achieved in pure water (see Supplementary Information Table S19) [72]. Conversely, in DESs, particularly ChCl-based DESs, the predominant product was the (R)-alcohol, ranging from 18.1 % ee to 88.7 % ee. This selectivity increased with decreasing water content. The highest selectivity was observed in ChCl:Glu (88.7 % ee). Similarly, the reduction of 1-(3,4-dimethylphenyl) ethenone using carrot root in water resulted in high enantioselectivity for the (S)-alcohol (95.6 % ee). However, in the presence of DESs (ChCl:Glu), the (R)-enantiomer becomes dominant (see Supplementary Information Table S15) [64]. Additionally, in the pH range from 3.9 to 6.0 of the medium, (R)-alcohol was prominent, whereas at other pH values the (S)-alcohol was favoured. Based on these studies it was argued that, the preferential formation of (R)-enantiomer was either due to the inhibitory action of the DES on cell ADHs (towards Prelog ADH; see Section 1.1) or due to the altered pH of the medium [64].

Utilising the same DES and whole cells, it is possible to synthesise different enantiomers with different substrates. For example, with the same ADH source strain, *G. geotrichum* ZJPH1810, different enantiomers were obtained by maintaining the DES, B: Lys constant. For the substrate 2,6-dichloro-3-fluoroacetophenone, the (S)-alcohol was obtained predominantly (>99.9 % ee) in B: Lys while for 4-(trifluoromethyl) acetophenone, the (R)-alcohol was the major product (63.7 % ee) (see Supplementary Information Tables S20 and S21) [92]. This indicates the influence of substrate as suggested by the Keinan model (see Fig. 4b above) [56].

**Scheme 7:**
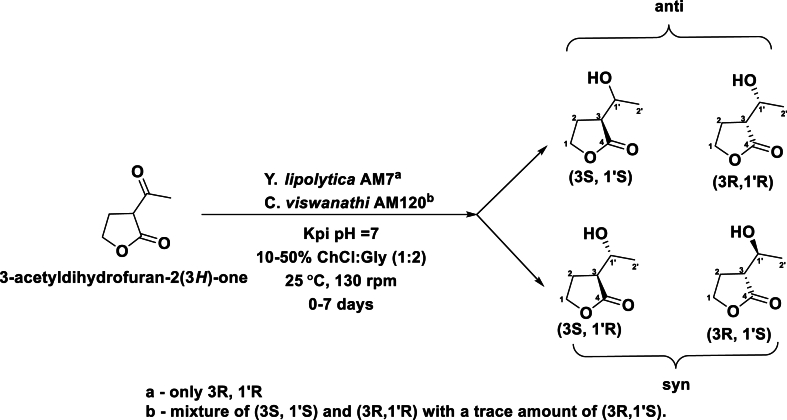
Asymmetric reduction of 3-acetyldihydrofuran-2(3H)-one with *Y.lipolytica* AM7 and *C.viswanathi* AM120. Kpi is potassium phosphate.

The stereochemistry of different ketone derivatives were also observed to be tunable with RasADH (overexpressed in *E.coli)* with different DES compositions, ChCl:Gly (1:1.5), ChCl:U (1:2) and ChCl:EG (1:2). The highest yield was obtained in ChCl:Gly [(S)-alcohol)] and the ee ratio increased with the use of DES even at 90 % (v/v; >95 % ee) (see Supplementary Information Table S16), but a low ee was obtained without DES [100]. Extending this work, different ADH activities from yeast, three strains of *Yarrowia lipolytica* (AM71, AM72, P26A)*, Candida viswanathi* AM120, *Hansenula anomala* C2, *Saccharomyces cerevisiae* K1, and *Saccharomyces pombe* C1 were investigated for the reduction of α-butyrolactone (see Scheme 7) [63]. Among these, the *Yarrowia* strains reduced the α-butyrolactone most efficiently, but yielded the *anti*-diastereomer; *Candida viswanathi* AM120 reduced the substrate with less stereoselectivity, and no substrate transformation was observed with *Saccharomyces*. *Yarrowia lipolytica* AM71 converted the α-butyrolactone into its corresponding (3R,1ˈR) product and *Candida viswanathi* AM120 converted the substrate into mixture of (3S, 1ˈS) and (3R,1ˈR) with a trace amount of (3R,1ˈS).

This suggests that enantioselectivity depends on DES, the whole cell involved and the nature of the substrate.

The concept of solvent control can be applied in this context, where altering the DES composition can induce stereo inversion. ChCl:Gly can be considered as the first option DES for ADH-based reduction of carbonyl compounds and enantioselective reactions [58,59,63,64,72,74,79,83,100,106]. The mechanism behind stereo inversion may involve the inhibition of (R) or (S) selective ADH enzymes present in the whole cells, although the mechanistic details are yet to be proven. Conversely, acidic DESs have demonstrated lower efficiency in biocatalysis [83]. Hence, the pivotal element in biocatalysis is the meticulous choice of DESs and the water/buffer:DES composition [83]. These two factors stand out as the primary optimisation parameters in the solvent system. Following these, additional molecules such as surfactants, which enhance substrate solubility, come into play [75,76]. Subsequently, NADES have proven to be more efficient for whole cell-based reduction reactions since they can provide a more suitable environment for the chosen whole cell [92].

Despite several advantages, the use of whole cells pose various operational challenges such as byproduct formation, difficulty in product isolation, and substrate/product toxicity to the cells [49]. In many cases, using isolated enzymes may be a more convenient arrangement, both in terms of handling, operation and product separation.

## 3 Isolated ADH-based reductions of carbonyl compounds in deep eutectic solvents

**Scheme 8:**
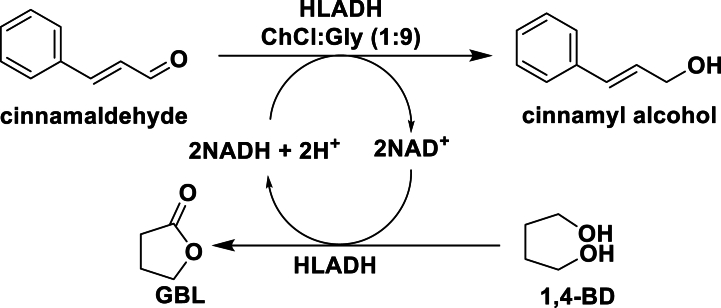
Asymmetric reduction of cinnamaldehyde with HLADH in ChCl:Gly.

The use of isolated enzymes in DES-buffer systems for bioreduction offers a solution to some of the barriers presented by whole cell enzyme sources. Isolated ADH was used in the reduction of different aryl prochiral ketones into their corresponding alcohols [24]. Interestingly, in the conversion of propiophenone to 1-phenyl-1-propanol, with the Codex® ADH screening kit, the isolated enzyme was inactive in ChCl:U and ChCl:LA DESs systems; whereas it showed activity in ChCl:Gly and ChCl:sorbitol (Sor). Certain ADHs retained activity with increasing DES concentration from 50 % to 80 % (w/w), suggesting this solvent system is a suitable environment for biocatalysis. Significantly in this example, the use of DESs led to a notable improvement in ee of the final product, indicating the influential role of DESs in enantioselectivity (see Supplementary Information Table S22) [24]. Furthermore, the water content and the components of DESs, especially HBDs, can play a significant role in determining the reaction yield for isolated ADH-based reductions. For example, the most efficient bioreduction of cinnamaldehyde to cinnamyl alcohol was achieved at a higher HBA:HBD ratio of 1:9 in ChCl:Gly using HLADH (see Scheme 8) [25].

**Fig. 5:**
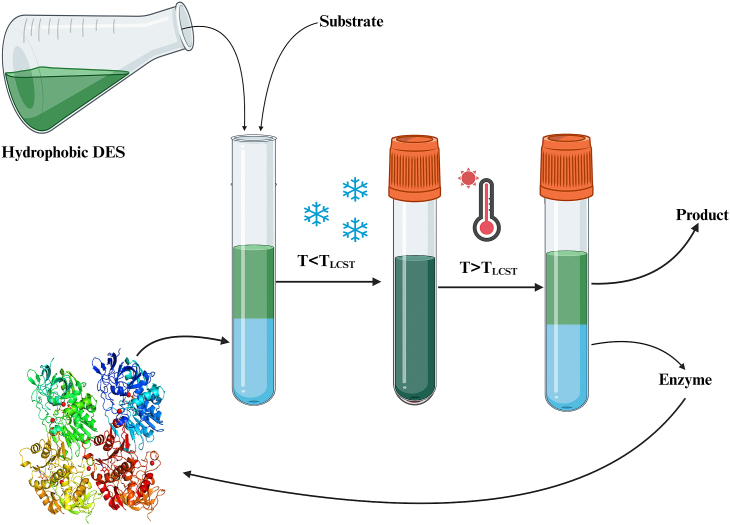
Schematic illustraiton of thermomorphic multiphase system (TMS). T is the system temperature, T_LCST_ is the lower critical solution temperature. Created with BioRender.com.

An interesting approach to simplify product recovery involves a hydrophobic DES with aqueous buffer as a two-phase system. The strategy employs a thermomorphic multiphase system (TMS). In this arrangement, the reaction takes place when the buffer, containing the enzyme, and the DES [lidocaine:oleic acid (L:Ole); 1:1)], containing the substrate, are brought into a single-phase at lower temperature (<lower critical solution temperature). This step is followed by increasing the temperature (>lower critical solution temperature) to re-form the two-phase regime such that the enzyme associates with the aqueous phase and the substrate/products self-isolate into the hydrophobic phase (see Fig. 5). The major advantage of using this system is the separation of product and ease of enzyme recovery (see Supplementary Information Table S23) [26].

In another application, a series of halogenated ketones can be reduced by lyophilised *E. coli* ADH [(R)-selective ADH from *Lactobacillus brevis* (*Lb*ADH) and (S)-selective ADHs from *Rhodococcus ruber* (ADH-A); Supplementary Information Table S24] [107].

The utilisation of isolated enzymes is constrained by the requirement for expensive cofactors. Therefore, it is essential to explore and devise strategies for efficient cofactor regeneration (see section 4 below).

## 4 Cofactor regeneration in deep eutectic solvents

**Fig. 6:**
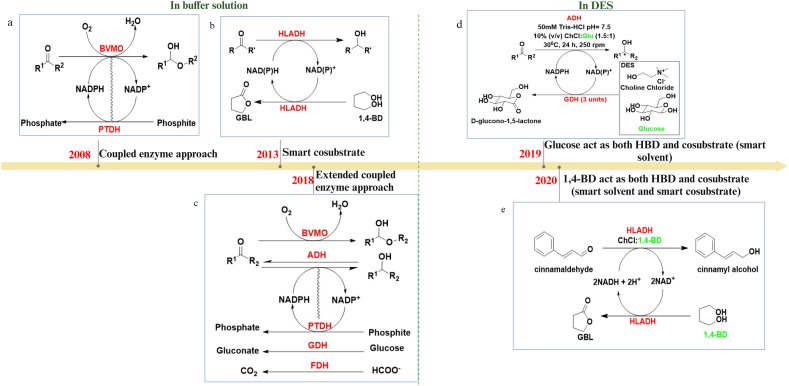
Overview of cofactor regeneration strategies over time with the introduction of DES in ADH catalysed reactions. a) Coupled enzyme approach in which the oxidation of phosphite by PTDH facilitates cofactor regeneration [110]. b) Smart cosubstrate, in which the 1,4-BD is getting oxidised to thermodynamically irreversible and kinetically inert coproduct facilitating the cofactor regeneration [108]. c) Extended coupled enzyme, in which PTDH, GDH, FDH are used for cofactor regeneration by oxidising phosphite, glucose, formate respectively [111]. d) smart solvent, in this case, glucose acts as HBD in DES and as a cosubstrate in the enzyme reaction for cofactor regeneration [112]. e) smart solvent and smart cosubstrate, 1,4-BD acts as HBD in the DES as well as being oxidised to a corresponding thermodynamically irreversible and kinetically inert coproduct [109]. The central yellow arrow denotes time. Abbreviations: BVMO is Baeyer-Villiger monooxygenases, HLADH is horse liver alcohol dehydrogenase, PTDH is phosphite dehydrogenase, GDH is glucose dehydrogenase and FDH is formate dehydrogenase. Created with BioRender.com.

Cofactors play a crucial role in redox biocatalysis in general, and ADH-based reactions specifically. As they can be expensive (See Supplementary Information Table S25), auto regeneration within the reaction system is a key requirement for a sustainable bioprocess. This can be achieved by carefully designing a co-reaction to regenerate the cofactor. The regeneration of a cofactor requires the addition of a cosubstrate. The cosubstrate will get oxidised (e.g. *iso*-propanol to acetone) and the cofactor will get reduced (e.g. NAD^+^ to NADH) accomplishing *in-situ* regeneration of cofactor. In some instances, the cosubstrate can also influence the conversion of substrate negatively. In the case of the reduction of propiophenone to (S)-1-phenylpropan-1-ol by using isolated RasADH in ChCl:Gly (1:2), the addition of propan-1-ol and propan-2-ol as cosubstrates enhanced the reactivity, whereas ethanol hampered the conversion [100]. One of the advancements in this area is the introduction of a smart cosubstrate. 1,4-BD was added as the cosubstrate in the reduction of various ketones in presence of Tris-HCl buffer (pH 7.0) with ADH. The 1,4-BD was oxidised to γ-butyrolactone (GBL) which is a thermodynamically irreversible and kinetically inert coproduct [108]. An advancement in this concept is the use of HLADH for the conversion of cyclohexanone to cyclohexanol; 1,4-BD in the DES, ChCl:1,4-BD acts as the cosubstrate, resulting in a more sustainable bioprocess. Furthermore, 1,4-BD acts as a smart cosubstrate, as it becomes irreversibly oxidised to a coproduct GBL (See Fig. 6) [109].

**Fig. 7:**
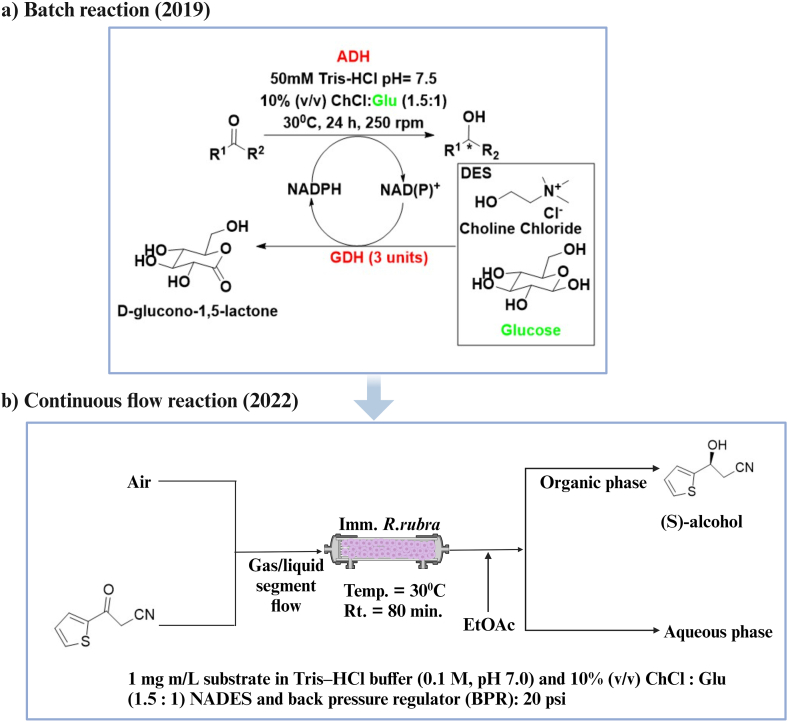
Advancement of the smart solvent concept from batch reaction to continuous flow reaction in ADH catalysed reactions. (a) Batch reaction in which glucose acts as both HBD of DES and as a cosubstrate for cofactor regeneration [112]. (b) Continuous flow system with the concept of smart solvent for the reduction of 3-oxo-3-(thiophen-2-yl)propanenitrile [113]. Imm. *R. rubra* is immobilised whole cells of *Rhodotorula rubra* MIM14. Created with BioRender.com.

A coupled enzyme reaction concept was developed as an efficient approach for the regeneration of a cofactor. For example, the Baeyer-Villiger monooxygenases (BVMO), coupled with NADPH-regenerating phosphite dehydrogenase (PTDH), was employed for a sustainable reduction reaction (see Fig. 6) [110]. This approach was further extended by coupling BVMO and ADH with regenerating enzymes like PTDH, glucose dehydrogenase (GDH) and formate dehydrogenase (FDH). The best result was obtained from the PTDH coupled enzyme as the oxidation of phosphite is irreversible [111]. These reactions were conducted in an aqueous buffer solution (Tris-HCl, pH 7.5) and, in this arrangement, the cosubstrate (glucose, formate, phosphite) had to be externally added. When DES is used as a smart solvent, it avoids this need for external additions to the reaction mixture. In this arrangement, the DES can act as a solvent and the HBD component of the DES can act as a cosubstrate, thereby eliminating the need for external addition of cosubstrate. For example, when ChCl:Glu was used for the reduction of acetophenone using *Lb*ADH, there was no need to add glucose as cosubstrate for cofactor regeneration [112]. Instead, glucose present as the HBD in the DES is oxidised to D-glucono-1,5-lactone by GDH and, concomitantly, NADP^+^ is converted to NADPH, resulting in cofactor regeneration. This dual-purpose utilisation of the solvent and its components marked a significant advancement in cofactor regeneration techniques (see Fig. 6). This concept was initially investigated in batch reactions [112] and later translated to a continuous flow reactor (see Fig. 7) [113]. This novel use of DES, as an approach to remove the need for addition of external cosubstrate to achieve cofactor regeneration, underscores the efficiency and promiscuity of DESs as a redox reaction enhancing solvent.

## 5 Conclusion

Biocatalysis is evolving as a sustainable approach for generating products currently unattainable, or causing environmental damage, through conventional chemical synthesis. The transition from organic solvents to DES marked a breakthrough in this field. This review explored the benefits of incorporating DES in redox biocatalysis using whole cells or isolated enzymes. The use of DES in biocatalysis, in redox or other reactions, benefits from lower toxicity, biodegradability, and ease of preparation. Beyond these, DES-based biocatalysis can significantly enhance the stability, activity, and enantioselectivity of enzymes; making them powerful solvents in redox biocatalysis. DESs can be tailored, through judicious solvent engineering, to induce changes in reaction output. For instance, altering the DES composition in some reactions can change the product's configuration as well as enhancing enantioselectivity, enzyme activity and stability. Additionally, adjusting the molar ratio of HBD components can enhance reaction yield.

This review of the literature suggests ChCl:Gly as a useful starting point for ADH-based enantioselective carbonyl reduction reactions. ChCl:Gly is stable, as neither ChCl or Gly participate in the ADH reaction and which supports the hydrogen bonding between the components. The enantioselectivity observed for certain whole-cell reactions in the presence of DES, especially in ChCl:Gly, is understood to be as a result of the inhibitory action of (R) or (S) selective ADH, although this concept lacks mechanistic confirmation and requires further investigation.

Looking to the future of redox biocatalysis in DES, a major challenge is the involvement of expensive cofactors. Current research focusses on extending the concepts of smart solvent and smart cosubstrate as potential solutions and these illuminate the path for future sustainable development. In certain reactions, the HBD of DES (e.g., Glu in ChCl:Glu, 1,4-BD in ChCl:1,4-BD) can serve both as a component in DES and as a cosubstrate for cofactor regeneration. This eliminates the need for external addition of cosubstrate for cofactor regeneration which is the concept of smart solvent. The smart cosubstrate concept involves the irreversible oxidation of the cosubstrate to a kinetically inert coproduct, leading to cofactor regeneration (eg: oxidation of 1,4-BD to GBL). Both concepts have been successfully applied in ChCl:1,4-BD and the concept of smart solvent has been adopted to continuous flow reactions, marking a milestone in biocatalysis. Extending, and scaling up, these fundamental concepts will provide the next steps for researchers working towards developing sustainable redox biocatalytic systems.

**Table:** Abbreviations

ADH	Alcohol dehydrogenase
1,4-BD	1,4-butanediol
B	Betaine
BTAP	1-(3,5-dimethylphenyl)ethan-1-one
BTPE	1-(3,5-dimethylphenyl)ethan-1-ol
BVMO	Baeyer-Villiger monooxygenases
BY	Baker's Yeast
C	l-Carnitine
CD	Cyclodextrin
C_4_MIM·PF_6_	1-butyl-3-methylimidazolium hexafluorophosphate
ChCl	Choline chloride
ChAc	Choline acetate
Cys	Cysteine
CFPL	2-chloro-1-(3,4-difluorophenyl)ethanol
CFPO	2-chloro-1-(3,4-difluorophenyl)ethenone
CHBE	4-chloro-3-hydroxybutanoate
COBE	Ethyl 4-chloro-3-oxobutanoate
CPE	2-chloro-1-(2,4-dichlorophenyl)ethanone
CPEO	2-chloro-1-(2,4-dichlorophenyl)ethanol
DES	Deep eutectic solvents
ee	Enantiomeric excess
EHB	Ethyl 3-hydroxybutyrate
EG	Ethylene glycol
EAA	Ethyl acetoacetate
F	Fructose
FDH	Formate dehydrogenase
FDM	Furandimethanol
G	Glycine
GBL	γ-butyrolactone
GDH	Glucose dehydrogenase
Gly	Glycerol
Glu	Glucose
GSH	Glutathione
HAP	Hydroxyacetophenone
HLADH	Horse liver alcohol dehydrogenase
HBA	Hydrogen bond acceptors
HBD	Hydrogen bond donors
IL	Ionic liquids
KRED	Ketoreductases
L	Lidocaine
LA	Lactic acid
LBADH	*Lactobacillus brevis* alcohol dehydrogenase
Lys	Lysine
MA	Malic acid
MCD	Methylated-β-cyclodextrin
MOAP	4′-methoxyacetophenone
MOPE	1-(4-methoxyphenyl)ethanol
NADES	Natural deep eutectic solvents
NADPH	Nicotinamide adenine dinucleotide phosphate hydrogen
OA	Oxalic acid
P	l-Proline
PED	1-phenyl-1,2-ethanediol
PBS	Phosphate buffered saline
PTDH	Phosphite dehydrogenase
ADH-A	*Rhodococcus ruber*ADH
RasADH	*Ralstonia* sp. ADH
Sor	Sorbitol
TMS	Thermomorphic multiphasic system
TesADH	*Thermoanaerobacter ethanolicus* ADH
Tre	Trehalose
TEG	Triethylene glycol
U	Urea

## Data availability

All the relevant data are included in the manuscript and the supplementary document. No separate repository is attached.

## CRediT authorship contribution statement

**Ebin K. Baby:** Writing – original draft. **Rangasamy Savitha:** Writing – review & editing. **Gemma K. Kinsella:** Writing – review & editing, Funding acquisition. **Kieran Nolan:** Writing – review & editing, Funding acquisition. **Barry J. Ryan:** Writing – review & editing, Funding acquisition. **Gary T.M. Henehan:** Writing – review & editing, Funding acquisition.

## Declaration of competing interest

The authors declare the following financial interests/personal relationships which may be considered as potential competing interests: Prof. Gary Henehan reports financial support was provided by 10.13039/501100001602Science Foundation Ireland. Prof. Gary Henehan reports a relationship with Technological University Dublin that includes: employment. If there are other authors, they declare that they have no known competing financial interests or personal relationships that could have appeared to influence the work reported in this paper.
