# Supplementary material for: Influence of deep eutectic solvents on redox biocatalysis involving alcohol dehydrogenases
Source: Heliyon. 2024 Jun 6;10(12):e32550. doi: 10.1016/j.heliyon.2024.e32550 (PMC11209023; doi:10.1016/j.heliyon.2024.e32550)
Supplement: Multimedia component 1 [file mmc1.docx]

**Supplementary Information**

**Influence of deep eutectic solvents on redox biocatalysis involving alcohol dehydrogenases**

Ebin K. Baby^1^, Rangasamy Savitha^1^, Gemma K. Kinsella^1^, Kieran Nolan^2^, Barry J. Ryan^1^, Gary T. M. Henehan^1^

^1^School of Food Science and Environmental Health, Technological University Dublin, Grangegorman Lower, Dublin 7, D07 E244.

^2^School of Chemical Sciences, Dublin City University, Glasnevin, Dublin 9, D09 V209

Please note the references in the supplemental are given at the end of this document.





**Fig. S1** Progress in solvent systems: characteristics, benefits, drawbacks, synthesis, and Illustrative examples

Table S1 Asymmetric reduction of 2-octanone with Acetobacter pasteurianus Gim1.158 cell in IL-DES system, yield and application of the product

| **Reaction** | | |
| --- | --- | --- |
|  | | |
| **Yield**  **(%)** | **Application of product** | **Ref.** |
| 95.7 | Applications in flavors and fragrances, paints and coatings, inks, adhesives, home care, lubricants and fuels | [1] |

Table S2 Asymmetric reduction of 2-hydroxy-1-phenylethan-1-one with K. gibsonii SC0312 cells, yield and application of the product

| **Reaction** | | |
| --- | --- | --- |
|  | | |
| **Yield**  **(%)** | **Application of product** | **Ref.** |
| 80 | Building block for acquiring enantiopure medications, including β-adrenergic blocking agents, designed for the treatment of cardiovascular disease and sympathetic nervous system disorders. | [2] |

Table S3 Asymmetric reduction of 3,5-BTAP with T. asperellum ZJPH0810, Yield of the reaction and the application of the product

| **Reaction** | | | | | |
| --- | --- | --- | --- | --- | --- |
|  | | | | | |
| **Medium** | **DES (%w/v)** | **Yield**  **(%)** | **ee**  **(%)** | **Application of product** | **Ref.** |
| ChCl:Glu(1:2) | 0.5 | 57.8 ± 1.3 | >99 | Production of antiemetic medications, specifically aprepitant and fosaprepitant. employed for the prevention of chemotherapy side effects. | [3] |
| ChCl:Glu(1:2) | 1 | 66.0 ± 0.8 | >99 |  |  |
| ChCl:Glu(1:2) | 1.5 | 60.7 ± 0.9 | >99 |  |  |
| ChCl:Cys(1:1) | 0.5 | 56.9 ± 0.8 | >99 |  |  |
| ChCl:Cys(1:1) | 1 | 64.1 ± 2.6 | >99 |  |  |
| ChCl:Cys(1:1) | 1.5 | 54.7 ± 1.2 | >99 |  |  |
| ChCl:Cys(1:2) | 0.5 | 62.4 ± 1.9 | >99 |  |  |
| ChCl:Cys(1:2) | 1 | 80.7 ± 1.3 | >99 |  |  |
| ChCl:Cys(1:2) | 1.5 | 70.5 ± 1.1 | >99 |  |  |
| ChCl:G(1:1) | 0.5 | 56.2 ± 0.8 | >99 |  |  |
| ChCl:G(1:1) | 1 | 63.1 ± 1.4 | >99 |  |  |
| ChCl:G(1:1) | 1.5 | 60.4 ± 1.3 | >99 |  |  |
| ChCl:G(1:2) | 0.5 | 54.2 ± 1.2 | >99 |  |  |
| ChCl:G(1:2) | 1 | 61.3 ± 0.9 | >99 |  |  |
| ChCl:G(1:2) | 1.5 | 60.6 ± 1.4 | >99 |  |  |
| ChCl:GSH(1:0.5) | 0.5 | 81.7 ± 0.5 | >99 |  |  |
| **ChCl:GSH(1:1)** | **1** | **90.7 ± 0.6** | **>99** |  |  |
| ChCl:GSH(1:2) | 1.5 | 78.3 ± 0.9 | >99 |  |  |

Table S4 Asymmetric reduction of FPOPA with Candida parapsilosis ZJPH1305, yield of the reaction and application of the product

| **Reaction** | | | | | |
| --- | --- | --- | --- | --- | --- |
|  | | | | | |
| **Medium** | **Yield**  **(%)** | **ee**  **(%)** | **Time**  **(h)** | **Application of product** | **Ref.** |
| Aqeous buffer | 65.9 | >99 | 72 | Chiral intermediate utilised in the manufacturing process of the cholesterol-lowering medication Ezetimibe. | [3] |
| ChCl:GSH-buffer | 83.8 | >99 | 72 |  |  |

Table S5 Asymmetric reduction of EAA with recombinant E. coli, yield of the reaction and application of the product

| **Reaction** | | | | | |
| --- | --- | --- | --- | --- | --- |
|  | | | | | |
| **Medium** | **Time (hour)** | **Yield**  **(%)** | **ee**  **(%)** | **Application of product** | **Ref.** |
| Aqeous buffer | 1.5 | 99.9 | >99 | Intermediate in the synthesis of (*R*)-3-hydroxybutyl-(*R*)-3-hydroxybutyrate, an ergogenic ketone body ester. | [3] |
| ChCl:GSH-buffer | 1 | 99.9 | >99 |  |  |

Table S6 Asymmetric reduction of BTAP with cberlindnera saturnus ZJPH1807, yield of the reaction and the application of the product

| **Reaction** | | | |
| --- | --- | --- | --- |
|  | | | |
| **Yield**  **(%)** | **ee**  **(%)** | **Application of product** | **Ref.** |
| 81 | >99 | Intermediate used in the synthesis of aprepitant, a representative NK-1 receptor antagonist known to alleviate nausea and vomiting induced by chemotherapy. | [4] |

Table S7 Asymmetric reduction of COBE with recombinant E. coli CCZU-T15 cells, yield of the reaction and the application of the product

| **Reaction** | | | |
| --- | --- | --- | --- |
|  | | | |
| **Yield**  **(%)** | **ee**  **(%)** | **Application of product** | **Ref.** |
| 91 | >99 | A chiral precursor for synthesising cholesterol-lowering hydroxymethylglutaryl-CoA reductase inhibitors like atorvastatin calcium. | [5] |

Table S8 Asymmetric reduction of 2-chloro-1-(2,4-dichlorophenyl)ethenone with Cyberlindnera saturnus ZJPH1807, yield, ee of the reaction, concentration of substrate, concentration of MCD and the application of the product

| **Reaction** | | | | | | |
| --- | --- | --- | --- | --- | --- | --- |
|  | | | | | | |
| **Medium** | **Substrate concentration (mM)** | **Concentration of MCD**  **(mM)** | **Yield**  **(%)** | **ee**  **(%)** | **Application of product** | **Ref.** |
| Phosphate buffer | 35.8 | 0 | 58.51.5 | >99.2 | Chiral precursor for the synthesis of miconazole, an antifungal drug known for its broad antibacterial spectrum, low toxicity, and low bacterial resistance | [6] |
| C:Tre + MCD + buffer | 44.7 | 0 | 50.6 ± 1.2 | *>*99.2 |  |  |
|  | 67.1 | 0 | 39.6 ± 2.1 | *>*99.2 |  |  |
|  | 35.8 | 80 | 91 ± 1.9 | *>*99.2 |  |  |
|  | 44.7 | 90 | 86.1 ± 1.6 | *>*99.2 |  |  |
|  | 67.1 | 100 | 84.2±2.0 | *>*99.2 |  |  |

Table S9 Asymmetric reduction of different ketones with saccharomyces cerevisiae in different DES and theconversion of the reaction. a; 1-(3-methylphenyl)ethanone, b; 1-(3,4-dimethylphenyl)ethenone and c; 1-(2,4,6-trimethyphenyl)ethenone

| **Reaction** | | | | |
| --- | --- | --- | --- | --- |
|  | | | | |
| **Substrate** | **DES** | **Conversion**  **(%)** | **ee**  **(%)** | **Ref.** |
| MPA^a^ | Pure water | 69.3 ± 4.9 | 30.0 ± 4.6 (S) | [8] |
|  | ChCl:Glu +80% (v/v) | 96.4 ± 4.0 | 20.3 ± 3.3 (S) |  |
|  | ChCl:EG +80% (v/v) | 89.1 ± 0.9 | 17.5 ± 2.5 (S) |  |
|  | ChCl:Gly +30% (v/v) | 82.1 ± 1.0 | 36.4 ± 3.6 (S) |  |
|  | ChCl:Gly +50% (v/v) | 85.0 ± 1.3 | 30.0 ± 3.0 (S) |  |
|  | ChCl:Gly +80% (v/v) | 91.0 ± 2.8 | 29.3 ± 2.9 (S) |  |
| DMPA^b^ | Pure water | 73.4 ± 0.5 | 68.1 ± 1.9 (S) |  |
|  | ChCl:Glu +80% (v/v) | 89.8 ± 3.5 | 61.2 ± 3.1 (S) |  |
|  | ChCl:EG +80% (v/v) | 91.8 ± 1.1 | 84.9 ± 0.1 (S) |  |
|  | ChCl:Gly +30% (v/v) | 75.1 ± 4.3 | 93.3 ± 2.5 (S) |  |
|  | ChCl:Gly +50% (v/v) | 86.4 ± 2.5 | 77.4 ± 1.1 (S) |  |
|  | ChCl:Gly +80% (v/v) | 91.4 ± 0.4 | 52.9 ± 2.8 (S) |  |
| TMPA^c^ | Pure water | 46.2 ± 1.6 | 56.0 ± 0.2 (S) |  |
|  | ChCl:Glu +80% (v/v) | 85.8 ± 3.0 | 67.8 ± 0.2 (S) |  |
|  | ChCl:EG +80% (v/v) | 75.5 ± 3.0 | 74.0 ± 4.4 (S) |  |
|  | ChCl:Gly +30% (v/v) | 44.9 ± 4.4 | 84.3 ± 6.7 (S) |  |
|  | ChCl:Gly +50% (v/v) | 63.0 ± 6.3 | 73.0 ± 3.2 (S) |  |
|  | ChCl:Gly +80% (v/v) | 85.5 ± 6.5 | 68.7 ± 3.8 (S) |  |

Table S10 Asymmetric reduction of 2-chloro-1-(3,4-difluorophenyl)ethanone with recombinant E. coli cells, yield of the reaction, enantiomeric excess and the application of the product

| **Reaction** | | | |
| --- | --- | --- | --- |
|  | | | |
| **Yield**  **(%)** | **ee**  **(%)** | **Application of product** | **Ref.** |
| 87 | >97 | A chiral building block employed in the synthesis of the P2Y_12_ receptor antagonist Ticagrelor (Brilinta®), a medication utilised in the treatment of acute coronary syndromes | [9] |

Table S11 Asymmetric reduction of CFPO with recombinant E. coli containing NADH-dependent reductase CmCR, yield of the reaction, enantiomeric excess and the application of the product

| **Reaction** | | | |
| --- | --- | --- | --- |
|  | | | |
| **Yield**  **(%)** | **ee**  **(%)** | **Application of product** | **Ref.** |
| 95.9 | >97 | Chiral building block utilised in the production of ticagrelor | [10] |

Table S12 Asymmetric reduction of 2,6-dichloro-3-fluoroacetophenone with G.geotrichum ZJPH1810, yield, ee of the reaction and the application of the product

| **Reaction** | | | | |
| --- | --- | --- | --- | --- |
|  | | | | |
| **Medium** | **Yield**  **(%)** | **ee**  **(%)** | **Application of product** | **Ref.** |
| Phosphate buffer | 63.4 | 99.9 (*S*) | Intermediate used in the synthetic preparation of Crizotinib, a powerful and selective dual inhibitor targeting mesenchymal-epithelial transition factor (c-MET) kinase and anaplastic lymphoma kinase (ALK) | [11,12] |
| B:Glu:Gly | 65.4 | 99.9 (*S*) |  |  |
| B:Lys | 68.3 | 99.9 (*S*) |  |  |
| C:Lys | 71.1 | 99.9 (*S*) |  |  |

Table S13 Asymmetric reduction of 4-(trifluoromethyl)acetophenone with G. geotrichum ZJPH1810, yield of the reaction, enantiomeric excess and the application of the product

| **Reaction** | | | | |
| --- | --- | --- | --- | --- |
|  | | | | |
| **Medium** | **Yield**  **(%)** | **ee**  **(%)** | **Application of product** | **Ref.** |
| Phosphate buffer | 72.2 | 43.0 (*R*) | A chiral intermediate found in numerous pharmaceuticals, including the chemokine CCR5 antagonist used extensively in AIDS  treatment, DP1 receptor antagonist, and the fungicide *Econazole*. | [11] |
| B:Glu:Gly | 77.6 | 45.4 (*R*) |  |  |
| B:Lys | 78.5 | 63.7 (*R*) |  |  |
| C:Lys | 77.5 | 64.9 (*R*) |  |  |

Table S14 Asymmetric reduction of 4-(trifluoromethyl)acetophenone with recombinant E.coli, yield, ee of the reaction and the application of the product

| **Reaction** | | | |
| --- | --- | --- | --- |
|  | | | |
| **Yield**  **(%)** | **ee**  **(%)** | **Application of product** | **Ref.** |
| 92.4 | >99 | A chiral intermediate found in numerous pharmaceuticals, including the chemokine CCR5 antagonist used extensively in AIDS treatment, DP1 receptor antagonist, and the fungicide *Econazole*. | [11] |

Table S15 Enantioselective reduction of 1-(3,4-dimethylphenyl)ethanone with carrot root in different medium and corresponding enantiomeric excess

| **Reaction** | | | |
| --- | --- | --- | --- |
|  | | | |
| **Medium** | **ee**  **(%)** | | **Ref.** |
|  | R | S | [18] |
| Pure water | - | 95.6 |  |
| ChCl:EG + 30% water | 73 | - |  |

Table S16 Enantioselective reduction of propiophenone with RasADH in different medium and corresponding enantiomeric excess

| **Reaction** | | | |
| --- | --- | --- | --- |
|  | | | |
| **Medium** | **ee**  **(%)** | | **Ref.** |
|  | R | S | [19] |
| ChCl:Gly 0% (v/v) | - | 4 |  |
| ChCl:Gly 10% (v/v) | - | 6 |  |
| ChCl:Gly 20% (v/v) | - | 8 |  |
| ChCl:Gly 40% (v/v) | - | 18 |  |
| ChCl:Gly 60% (v/v) | - | 30 |  |
| ChCl:Gly 80% (v/v) | - | 76 |  |
| ChCl:Gly 90% (v/v) | - | 90 |  |
| ChCl:Gly 95% (v/v) | - | 99 |  |
| ChCl:Gly 100% (v/v) | - | - |  |

Table S17 Enantioselective reduction of EAA with Baker's yeast in different medium and corresponding enantiomeric excess

| **Reaction** | | | |
| --- | --- | --- | --- |
|  | | | |
| **Medium** | **ee**  **(%)** | | **Ref.** |
|  | R | S | [14] |
| Pure water | - | 95 |  |
| ChCl:Gly | 95 | - |  |

Table S18 Enantioselective reduction of aryl alkyl ketone with Baker's yeast resting cell in different medium and corresponding enantiomeric excess

| **Reaction** | | | |
| --- | --- | --- | --- |
|  | | | |
| **Medium** | **ee**  **(%)** | | **Ref.** |
|  | R | S | [16] |
| Pure water | - | 98 |  |
| ChCl:Gly + 20 w% water | - | 98 |  |

Table S19 Enantioselective reduction of 1-(3,4-dimethylphenyl)ethanone with Beta vulgaris L. subsp. vulgaris in different medium and corresponding enantiomeric excess

| **Reaction** | | | |
| --- | --- | --- | --- |
|  | | | |
| **Medium** | **ee**  **(%)** | | **Ref.** |
|  | R | S | [17] |
| Pure water | - | 86 |  |
| ChCl:Glu + 30% (v/v) water | 88.7 | - |  |

Table S20 Enantioselective reduction of 2,6-dichloro-3-fluoroacetophenone with G.geotrichum ZJPH1810 in different medium and corresponding enantiomeric excess

| **Reaction** | | | |
| --- | --- | --- | --- |
|  | | | |
| **Medium** | **ee**  **(%)** | | **Ref.** |
|  | R | S | [11] |
| B:Lys | - | 99.9 |  |
| C:Lys | - | 99.9 |  |

Table S21 Enantioselective reduction of 4-(trifluoromethyl)acetophenone with recombinant E. coli in different medium and corresponding enantiomeric excess

| **Reaction** | | | |
| --- | --- | --- | --- |
|  | | | |
| **Medium** | **ee**  **(%)** | | **Ref.** |
|  | R | S | [11] |
| B:Lys | 63.7 | - |  |
| C:Lys | 64.9 | - |  |

Table S22 Conversion of propiophenone to 1-phenyl-1-propanol in presence of different ADH (Codex®) by using different DES-buffer compositions. a is the DES-buffer expressed in %w, Kpi is the potassium phosphate buffer.

|  | | | | | | | | |
| --- | --- | --- | --- | --- | --- | --- | --- | --- |
| **Entry** | **ADH** | **Conversion (%)** | | | | | |  |
|  |  | **ChCl:Gly** | | | **ChCl:Sor** | | | **Ref.** |
|  |  | **50%^a^ DES** | **80%^a^ DES** | **100%^a^ DES** | **50%^a^ DES** | **80%^a^ DES** | **100%^a^ DES** | [21] |
| 1 | NADH-110 | 39 | - | - | 42 | 25 | - |  |
| 2 | P1-A04 | >99 | 33 | - | >99 | 93 | - |  |
| 3 | P1-C01 | 94 | 7 | - | >99 | 95 | - |  |
| 4 | P1-H10 | >99 | - | - | >99 | 92 | - |  |
| 5 | P2-C11 | >99 | >99 | 50 | >99 | >99 | - |  |
| 6 | P2-D12 | 90 | 32 | - | >99 | 88 | - |  |
| 7 | P2-H07 | >99 | - | - | >99 | 82 | - |  |
| 8 | P3-B03 | 45 | - | - | 50 | 33 | - |  |
| 9 | P3-G09 | 42 | - | - | 45 | 35 | - |  |
| 10 | P3-H12 | 90 | - | - | 94 | 81 | - |  |
| 11 | LKADH (*L. kefiri*) | >99 | 93 |  | 87 | 81 | - |  |

Table S23 Asymmetric reduction of benzaldehyde with HLADH in TMS, yield of the reaction with the cosubstrate used and the application of the product

|  | **Reaction** | | |
| --- | --- | --- | --- |
|  |  | | |
| **Cosubstrate** | **Yield**  **(%)** | **Application of product** | **Ref.** |
| Ethanol | 99.6 | Utilised as a local anesthetic, particularly when combined with epinephrine, it serves as a solvent for dyes, enhancing the dyeing process for materials such as wool, nylon, and leather. Additionally, it acts as a precursor for various esters and ethers, finding applications in the soap, perfume, and flavor industries | [23–25] |
| Without ethanol | 98.5 |  |  |

Table S24 Bioreduction of different halogenated ketones using ADH-A and LBADH, conversion and enantiomeric excess of the reaction.

|  | | | | | |
| --- | --- | --- | --- | --- | --- |
| **Entry** | **Substrate** | **ADH** | **Conversion (%)** | **ee**  **(%)** | **Ref.** |
| 1 | 1 | ADH-A | >99 | >99 (R) | [26] |
| 2 |  | LBADH | >99 | >99 (S) |  |
| 3 | 2 | ADH-A | >99 | >99 (R) |  |
| 4 |  | LBADH | >99 | >99 (S) |  |
| 5 | 3 | ADH-A | >99 | >99 (R) |  |
| 6 |  | LBADH | >99 | >99 (S) |  |
| 7 | 4 | ADH-A | >99 | >99 (R) |  |
| 8 |  | LBADH | 86 | >99 (S) |  |
| 9 | 5 | ADH-A | 96 | >99 (R) |  |
| 10 |  | LBADH | 95 | >99 (S) |  |
| 11 | 6 | ADH-A | >99 | >99 (R) |  |
| 12 |  | LBADH | >99 | >99 (S) |  |
| 13 | 7 | ADH-A | >99 | >99 (R) |  |
| 14 |  | LBADH | 97 | 97 (S) |  |
| 15 | 8 | ADH-A | >99 | >99 (R) |  |
| 16 |  | LBADH | >99 | >99 (S) |  |

Table S25 Comparative prices of phosphorylated and non-phosphorylated nicotinamide cofactor in Ireland as of January 2024

| **Cofactor** | **Company** | **Price in €** |
| --- | --- | --- |
| NAD^+^ | Sigma-Aldrich | 104 per g |
|  | Thermofischer Scientific | 77.6 per g |
| NADH | Sigma-Aldrich | 230 per g |
|  | Thermofischer Scientific | 156 per g |
| NADP^+^ | Sigma-Aldrich | 1100 per g |
|  | Thermofischer Scientific | 428 per g |
| NADPH | Sigma-Aldrich | 3020 per g |
|  | Thermofischer Scientific | 867 for 500mg |

**Reference**

[1] P. Xu, P.-X. Du, M.-H. Zong, N. Li, W.-Y. Lou, Combination of deep eutectic solvent and ionic liquid to improve biocatalytic reduction of 2-octanone with Acetobacter pasteurianus GIM1.158 cell, Sci Rep 6 (2016) 26158. https://doi.org/10.1038/srep26158.

[2] F. Peng, Q.-S. Chen, F.-Z. Li, X.-Y. Ou, M.-H. Zong, W.-Y. Lou, Using deep eutectic solvents to improve the biocatalytic reduction of 2-hydroxyacetophenone to (R)-1-phenyl-1,2-ethanediol by Kurthia gibsonii SC0312, Molecular Catalysis 484 (2020) 110773. https://doi.org/10.1016/j.mcat.2020.110773.

[3] J. Li, P. Wang, Y.-S. He, Z.-R. Zhu, J. Huang, Toward Designing a Novel Oligopeptide-Based Deep Eutectic Solvent: Applied in Biocatalytic Reduction, ACS Sustainable Chem. Eng. 7 (2019) 1318–1326. https://doi.org/10.1021/acssuschemeng.8b04989.

[4] S. Bi, H. Liu, H. Lin, P. Wang, Integration of natural deep-eutectic solvent and surfactant for efficient synthesis of chiral aromatic alcohol mediated by Cyberlindnera saturnus whole cells, Biochemical Engineering Journal 172 (2021) 108053. https://doi.org/10.1016/j.bej.2021.108053.

[5] Y. Dai, B. Huan, H.-S. Zhang, Y.-C. He, Effective Biotransformation of Ethyl 4-Chloro-3-Oxobutanoate into Ethyl (S)-4-Chloro-3-Hydroxybutanoate by Recombinant E. coli CCZU-T15 Whole Cells in [ChCl][Gly]–Water Media, Appl Biochem Biotechnol 181 (2017) 1347–1359. https://doi.org/10.1007/s12010-016-2288-0.

[6] Y. Zhang, Z.-W. Duan, H.-Y. Liu, F. Qian, P. Wang, Synergistic promotion for microbial asymmetric preparation of (R)-2-chloro-1-(2,4-dichlorophenyl)ethanol by NADES and cyclodextrin, Molecular Catalysis 526 (2022) 112376. https://doi.org/10.1016/j.mcat.2022.112376.

[7] L. Xiong, X. Kong, H. Liu, P. Wang, Efficient biosynthesis of (S)-1-[2-(trifluoromethyl)phenyl]ethanol by a novel isolate Geotrichum silvicola ZJPH1811 in deep eutectic solvent/cyclodextrin-containing system, Bioresource Technology 329 (2021) 124832. https://doi.org/10.1016/j.biortech.2021.124832.

[8] M. Panić, D. Delač, M. Roje, I. Radojčić Redovniković, M. Cvjetko Bubalo, Green asymmetric reduction of acetophenone derivatives: Saccharomyces cerevisiae and aqueous natural deep eutectic solvent, Biotechnol Lett 41 (2019) 253–262. https://doi.org/10.1007/s10529-018-2631-3.

[9] Y. He, Q. Huang, P. Wang, Design and evaluation of novel bio‐based deep eutectic solvents for highly efficient bioproduction of chiral aryl alcohol, J of Chemical Tech & Biotech 95 (2020) 1980–1988. https://doi.org/10.1002/jctb.6386.

[10] L. Kong, B. Fan, Y.-C. He, Efficient whole-cell biosynthesis of (S)-2-chloro-1-(3,4-difluorophenyl)-ethanol from 2-chloro-1-(3,4-difluorophenyl) ethanone in a sustainable reaction system, Molecular Catalysis 550 (2023) 113570. https://doi.org/10.1016/j.mcat.2023.113570.

[11] N. Xia, L. Xiong, S. Bi, F. Qian, P. Wang, Development of biocompatible DES/NADES as co-solvents for efficient biosynthesis of chiral alcohols, Bioprocess Biosyst Eng 43 (2020) 1987–1997. https://doi.org/10.1007/s00449-020-02387-5.

[12] J.-Q. Qian, P.-C. Yan, D.-Q. Che, Q.-L. Zhou, Y.-Q. Li, A novel approach for the synthesis of Crizotinib through the key chiral alcohol intermediate by asymmetric hydrogenation using highly active Ir-Spiro-PAP catalyst, Tetrahedron Letters 55 (2014) 1528–1531. https://doi.org/10.1016/j.tetlet.2014.01.053.

[13] P. Xu, Y. Xu, X.-F. Li, B.-Y. Zhao, M.-H. Zong, W.-Y. Lou, Enhancing Asymmetric Reduction of 3-Chloropropiophenone with Immobilized *Acetobacter* sp. CCTCC M209061 Cells by Using Deep Eutectic Solvents as Cosolvents, ACS Sustainable Chem. Eng. 3 (2015) 718–724. https://doi.org/10.1021/acssuschemeng.5b00025.

[14] Z. Maugeri, P. Domínguez de María, Whole‐Cell Biocatalysis in Deep‐Eutectic‐Solvents/Aqueous Mixtures, ChemCatChem 6 (2014) 1535–1537. https://doi.org/10.1002/cctc.201400077.

[15] P. Vitale, V.M. Abbinante, F.M. Perna, A. Salomone, C. Cardellicchio, V. Capriati, Unveiling the Hidden Performance of Whole Cells in the Asymmetric Bioreduction of Aryl‐containing Ketones in Aqueous Deep Eutectic Solvents, Adv Synth Catal 359 (2017) 1049–1057. https://doi.org/10.1002/adsc.201601064.

[16] P. Vitale, F. Perna, G. Agrimi, I. Pisano, F. Mirizzi, R. Capobianco, V. Capriati, Whole-Cell Biocatalyst for Chemoenzymatic Total Synthesis of Rivastigmine, Catalysts 8 (2018) 55. https://doi.org/10.3390/catal8020055.

[17] D. Pavoković, K. Košpić, M. Panić, I. Radojčić Redovniković, M. Cvjetko Bubalo, Natural deep eutectic solvents are viable solvents for plant cell culture-assisted stereoselective biocatalysis, Process Biochemistry 93 (2020) 69–76. https://doi.org/10.1016/j.procbio.2020.03.020.

[18] M. Panić, M.M. Elenkov, M. Roje, M.C. Bubalo, I.R. Redovniković, Plant-mediated stereoselective biotransformations in natural deep eutectic solvents, Process Biochemistry 66 (2018) 133–139. https://doi.org/10.1016/j.procbio.2017.12.010.

[19] C.R. Müller, I. Lavandera, V. Gotor‐Fernández, P. Domínguez de María, Performance of Recombinant‐Whole‐Cell‐Catalyzed Reductions in Deep‐Eutectic‐Solvent–Aqueous‐Media Mixtures, ChemCatChem 7 (2015) 2654–2659. https://doi.org/10.1002/cctc.201500428.

[20] W. Mączka, K. Wińska, M. Grabarczyk, B. Żarowska, Yeast-Mediated Stereoselective Reduction of α-Acetylbutyrolactone, Applied Sciences 8 (2018) 1334. https://doi.org/10.3390/app8081334.

[21] L. Cicco, N. Ríos-Lombardía, M.J. Rodríguez-Álvarez, F. Morís, F.M. Perna, V. Capriati, J. García-Álvarez, J. González-Sabín, Programming cascade reactions interfacing biocatalysis with transition-metal catalysis in *Deep Eutectic Solvents* as biorenewable reaction media, Green Chem. 20 (2018) 3468–3475. https://doi.org/10.1039/C8GC00861B.

[22] J.P. Bittner, N. Zhang, L. Huang, P. Domínguez De María, S. Jakobtorweihen, S. Kara, Impact of deep eutectic solvents (DESs) and individual DES components on alcohol dehydrogenase catalysis: connecting experimental data and molecular dynamics simulations, Green Chem. 24 (2022) 1120–1131. https://doi.org/10.1039/D1GC04059F.

[23] L. Wilson, S. Martin, Benzyl Alcohol as an Alternative Local Anesthetic, Annals of Emergency Medicine 33 (1999) 495–499. https://doi.org/10.1016/S0196-0644(99)70335-5.

[24] M. Ash, I. Ash, Handbook of preservatives, Synapse Information Resources, Endicott, NY, 2009.

[25] L. Meyer, M.B. Andersen, S. Kara, Ein thermomorphes stark eutektisches Lösungsmittelmehrphasensystem für biokatalytische Anwendungen, Angewandte Chemie 134 (2022) e202203823. https://doi.org/10.1002/ange.202203823.

[26] F.Z. Ibn Majdoub Hassani, S. Amzazi, J. Kreit, I. Lavandera, Deep Eutectic Solvents as Media in Alcohol Dehydrogenase‐Catalyzed Reductions of Halogenated Ketones, ChemCatChem 12 (2020) 832–836. https://doi.org/10.1002/cctc.201901582.
